# Patient and caregiver perspectives on early identification for advance care planning in primary healthcare settings

**DOI:** 10.1186/s12875-020-01206-w

**Published:** 2020-07-09

**Authors:** Cynthia Kendell, Jyoti Kotecha, Mary Martin, Han Han, Margaret Jorgensen, Robin Urquhart

**Affiliations:** 1 Cancer Outcomes Research Program, Department of Surgery, Dalhousie University and Nova Scotia Health Authority, Halifax, Nova Scotia Canada; 2 Department of Family Medicine, Queen’s University, Kingston, Ontario Canada; 3 Department of Surgery, Dalhousie University, Halifax, Nova Scotia Canada; 4 Department of Community Health and Epidemiology, Dalhousie University, Halifax, Nova Scotia Canada

**Keywords:** Primary care, Advance care planning, End-of-life care, Electronic medical record

## Abstract

**Background:**

As part of a broader study to improve the capacity for advance care planning (ACP) in primary healthcare settings, the research team set out to develop and validate a computerized algorithm to help primary care physicians identify individuals at risk of death, and also carried out focus groups and interviews with relevant stakeholder groups. Interviews with patients and family caregivers were carried out in parallel to algorithm development and validation to examine (1) views on early identification of individuals at risk of deteriorating health or dying; (2) views on the use of a computerized algorithm for early identification; and (3) preferences and challenges for ACP.

**Methods:**

Fourteen participants were recruited from two Canadian provinces. Participants included individuals aged 65 and older with declining health and self-identified caregivers of individuals aged 65 and older with declining health. Semi-structured interviews were conducted via telephone. A qualitative descriptive analytic approach was employed, which focused on summarizing and describing the informational contents of the data.

**Results:**

Participants supported the early identification of patients at risk of deteriorating health or dying. Early identification was viewed as conducive to planning not only for death, but for the remainder of life. Participants were also supportive of the use of a computerized algorithm to assist with early identification, although limitations were recognized. While participants felt that having family physicians assume responsibility for early identification and ACP was appropriate, questions arose around feasibility, including whether family physicians have sufficient time for ACP. Preferences related to the content of and approach to ACP discussions were highly individualized. Required supports during ACP include informational and emotional supports.

**Conclusions:**

This work supports the role of primary care providers in the early identification of individuals at risk of deteriorating health or death and the process of ACP. To improve ACP capacity in primary healthcare settings, compensation systems for primary care providers should be adjusted to ensure appropriate compensation and to accommodate longer ACP appointments. Additional resources and more established links to community organizations and services will also be required to facilitate referrals to relevant community services as part of the ACP process.

## Background

The care that individuals receive at the end-of-life (EOL) does not always align with their values and preferences. For example, although many individuals would prefer to die at home [1, 2] and would prefer pain and symptom management (i.e., comfort care) over life-prolonging interventions [3, 4], the majority of deaths occur in hospital [5, 6]. Advance care planning (ACP) is one way to help ensure that patients receive care that is consistent with their values and preferences. ACP is a process wherein individuals with decision-making capacity reflect upon and express their wishes regarding their future health and/or personal care in the event that they become incapable of making decisions about their care [7, 8]. ACP is associated with a variety of benefits, including: care that aligns with patients wishes [9–11]; reduced stress, anxiety, and depression among surviving family members [9]; improved patient and family satisfaction with care [9, 12]; less aggressive care [11, 13, 14]; fewer in-hospital deaths [12]; and decreased healthcare costs [15, 16].

Unfortunately, suboptimal uptake of ACP has been reported across various populations [4, 10, 17–22]. Of particular concern is low uptake among patients experiencing chronic and potentially life limiting illness. For example, in a study of hospitalized cancer patients at a single institution in the United States, only 29% of the patients reported having had a discussion with their oncologist about their wishes if they became seriously ill or near death [20]. In the Netherlands, it was reported that of a sample of patients with severe chronic obstructive pulmonary disease (COPD) and congestive health failure (CHF), only 5.9% and 3.9% discussed advance directives with the physician specialist, respectively [18]. In a Canadian study examining ACP activities among hospitalized elderly who were deemed at risk of death within 6 months, less than half of patients (47.9%) reported having completed an advance care plan prior to their hospitalization [4]. Altogether, these findings highlight the need for an upstream approach to ACP.

There are various factors that contribute to low ACP uptake. One is that providers find it difficult to identify elderly patients who are at risk of dying in the near future [23, 24]. Even for individuals with a known chronic illness, a clear terminal phase does not always exist [25, 26]. This makes it difficult for physicians to determine an appropriate time to initiate discussions with patients about their prognosis or planning for EOL care [27–29]. Identification of those approaching the EOL often occurs too late for care plans to be put into place and carried out [24]. At the same time, patients and families recognize the importance of care planning for those who may be approaching the EOL [30, 31] but are often hesitant to approach the subject if their provider does not raise the issue first [30, 32–35].

Toward improving ACP uptake in Canada, we undertook a study with the overall objective of increasing the capacity (i.e., ability) of primary healthcare (PHC) providers and systems to engage in ACP for elderly patients who are frail and/or living with advanced life-limiting chronic illness. Primary care providers (PCPs) were the focus of this work for several reasons. First, they are well situated to take on a role in both early identification and in initiating the ACP process [36, 37]. Primary care providers are the point of first and continuing contact for patients, allowing them to respond to changes in health status in a timely manner, as well as build a rapport with patients that would be conducive to ACP. Second, most Canadians are seen by a PCP, specifically a family physician [38]. Third, a recent Canadian study of older primary care patients reported that only 52.8% had spoken to someone about their treatment preferences in the event of illness and hospitalization, and that only 17.5% of these conversations were with a family doctor (the vast majority spoke with family members) [22]. Thus, primary healthcare settings may benefit from the development and implementation of strategies and tools to improve capacity for ACP.

Our study included three separate components, or sub-studies: the development and validation of an algorithm using data captured in electronic medical records (EMRs) in PHC settings to support routine identification of individuals at risk of death in the next 12 months [39], focus groups and interviews with health professionals [40], and interviews with patients and patient caregivers (family member or friend) to learn about their perspective on the opportunities and challenges of identifying at-risk individuals and initiating ACP in PHC settings. Focus groups and interviews with relevant stakeholder groups were carried out in parallel to algorithm development and validation.

This paper focuses on the interviews with patients and caregivers. The specific objectives of this component of the study were to examine patient and caregiver (family/friend) (1) views on practice-level identification of individuals at risk of deteriorating health or dying ; (2) views on the use of an EMR- based algorithm for early identification in PHC settings; and (3) preferences and challenges for ACP in PHC settings.

## Methods

This qualitative descriptive study [41] used one-on-one, semi-structured interviews with patients and caregivers from Nova Scotia and Ontario.

### Participants

Participants included two groups of individuals: (1) patients of PCPs in Nova Scotia and Ontario, aged 65 and older, who were experiencing a decline in health, which was broadly defined to include loss of physical function, mild cognitive impairment, chronic illness, multi-morbidity, or some combination thereof, and (2) individuals who self-identified as being a caregiver of an individual aged 65 or older who was experiencing/had experienced a decline in health. Participants varied in terms of the nature and extent of decline they were experiencing, and their caregiving experiences. Inclusion criteria did not take into consideration whether individuals had previous experience with ACP. As such, the participants included in the study also had a wide range of familiarity and experiences with ACP. Individuals experiencing cognitive impairment that would affect their ability to provide informed consent were not included in the study.

### Recruitment

In Nova Scotia, participants were recruited from two Halifax-area clinical practices: a PHC practice and an orthopedic surgery follow-up clinic. Physicians identified eligible patients within their practice based on inclusion criteria provided by the research team. Patients who met the eligibility criteria were informed of the study by their physician, and if they were interested in participating they were referred to the Research Coordinator [CK] who was located on-site. The research coordinator provided a study information sheet and consent forms, answered questions, and confirmed individual interest. Those who confirmed interest in participating provided contact information so that they could be contacted to set up an interview. In some cases, patients were accompanied by family/friend caregivers who were invited to participate.

In Ontario, individuals were recruited from a seniors housing complex, a seniors living center, and the community. Individuals either self-identified, or were identified by members of the research team. A flyer containing study information and inclusion criteria and a sign-up sheet were placed in the seniors housing complex. Individuals who self-identified as meeting the inclusion criteria and could sign up to be contacted by the Research Coordinator [MM]. The manager of the seniors living center extended a personal, verbal invitation to members to participate in the study, and interested individuals consented to having their contact information shared with the research team. Individuals from the community who met the study criteria and were known to the research team also received a personal, verbal invitation to participate in the study.

### Data collection

In each province, data collection was carried out by a Research Coordinator with experience in qualitative methods [CK, MM and HH]. Data collection involved semi-structured interviews. The interview guide (Appendix A) was developed with guidance from both Patton [42] and Rubin and Rubin [43]. Consistent with our focus on early identification, and in an effort to prevent participants from focusing on the period of time immediately preceding death, we framed questions to participants in terms of being identified as “at risk of deteriorating health or dying”, rather than “end-of-life”. Advance care planning was described to participants as “the process by which a patient, in consultation with healthcare providers, considers options about future healthcare decisions and identifies his/her wishes and goals with respect to future health care”. Algorithm development and validation was in progress at the time of data collection. The algorithm was explained to participants who were then asked questions related to its potential usefulness and acceptability. All interviews were audio-recorded and transcribed verbatim.

### Data analysis

A qualitative descriptive approach was used [41]. Qualitative description is concerned with summarizing and describing the informational contents of the data. There is minimal interpretation and data are presented on their own terms—opinions are presented as opinions, preferences as preferences, and experiences as experiences. Qualitative description is “especially amenable to obtaining straight and largely unadorned (i.e., minimally theorized or otherwise transformed or spun) answers to questions of special relevance to practitioners and policy makers” (p.337).

Data were analyzed separately by the Nova Scotia and Ontario Research Coordinators [CK, MM and HH], with supervision from the provincial leads [RU and JK]. Analysis was performed manually and using Qualitative software (NVivo; QSR International, Cambridge, MA, USA). After initial coding of transcripts, the Research Coordinators and provincial leads met to review themes identified and refine codes. Disagreements were resolved through discussion amongst the Research Coordinators and provincial leads, with re-examination of transcripts and coded data as needed. Exemplary quotes were identified from within the data to illustrate key concepts and ideas.

Data collection and analysis continued until theoretical saturation was reached, so that no new substantive information was being collected. Preliminary analysis did not reveal substantive differences between findings in Ontario and Nova Scotia, so findings were aggregated through an iterative process of team discussions and reviewing interview data.

## Results

In total, 14 individuals were recruited (5 in Nova Scotia and 9 in Ontario; 3 male and 11 female). Of these, 3 individuals were caregivers and 11 were patients, although many individuals had experiences as both a patient and caregiver. This was especially the case for patients, many of whom had previously been the caregiver for a seriously ill spouse and/or parent. No patient-caregiver dyads participated in the study.

### Views on being identified as at risk of deteriorating health or death

In general, participants felt that early identification of patients who were at risk of deteriorating health or dying was a good idea and would be beneficial to patients. Specifically, early identification was regarded as beneficial for health-related planning (e.g., creating care directives, preparing a will, making funeral arrangements, moving to a nursing home, etc.) and enabling individuals to maintain personal autonomy. By having these plans in place, and communicating and formalizing their wishes, individuals felt it would reduce stress for both patients and their family members/caregivers. Moreover, by knowing they were at risk of deteriorating health or dying, individuals could focus on things like spending time with family or taking a long-awaited vacation. In other words, early identification allowed individuals to plan not only for death, but also for the remainder of life.

*“…it gave me my independence and respect and dignity as a human being that I did have a voice in the treatment towards the end of life care” [Ontario, Participant #5].*

“*The lawn still has to be mowed. The driveway has to be shoveled. The car has to have gas in it. The power bill has to be paid. You still need groceries. And it doesn’t matter if you’re dying tomorrow, all of those things are still somebody’s problem. And if they were dealt with at the time, it wouldn't be near as great a problem. So somebody has to start it.” [Nova Scotia, Participant #6]*

### Use of an EMR-based algorithm

Most participants were supportive of the use of an EMR-based algorithm to identify patients who are at risk of deteriorating health or death. An EMR-based algorithm was viewed as another tool available to physicians that could assist them in their clinical decision-making.

*“I personally think it’s a great idea. I don’t have a problem with it if it means that the doctor’s going to discover sooner that there’s an issue with my health.” [Ontario, Participant #8]*

However, there were a number of reservations expressed regarding this approach. The primary concern about the use of an algorithm was privacy. Specifically, participants were concerned that individuals other than their physician (e.g., insurance providers, staff at other facilities) would have access to the information generated by the algorithm.

*“I don’t see anything wrong about it but, uhm, again I guess I suppose computers, you are always wondering if this goes out to insurance companies and whatever.” [Nova Scotia, Participant #3]*

Participants also identified potential limitations of using an EMR-based algorithm in that such tools would not be applicable to patients without a primary care provider, all deaths cannot be predicted, and it would add to physicians’ workloads.

*“… I just can’t see it ever coming to be a real thing that’s ever going to be used. I don't think doctors have time for it.” [Nova Scotia, Participant #5]*

### Preferences for early identification and advance care planning

In terms of who should be identifying patients at risk of deteriorating health or death, and initiating and carrying out ACP, participants viewed PCPs (specifically, family physicians) as an appropriate choice because they have existing relationships with patients and would be more familiar with their specific circumstances. Moreover, most participants reported having good relationships with their family physicians and trusting them. One concern with relying on PCPs is that these conversations, which have the potential to be quite complex, may require more time than a PCP is typically able to spend with a patient.

*“I guess your doctor in the first place, and then with family, but the doctor would be the one to advise I would think, because he knows you, your situation. I really think it should be your family doctor who’s known you for probably, like with me, 37 years.” [Ontario, Participant #2]*

Participants typically agreed that these conversations should occur in a face-to-face manner, and that a separate visit should be dedicated to this conversation alone. Otherwise, there was substantial variation in terms of participant preferences. Specifically, participant views varied with regard to how the physician should approach the conversation, with some preferring a straightforward, candid approach, and others preferring a gentle approach, and some highlighting the need for communications training.

*‘…because the doctor has the knowledge but it doesn’t mean that he– some people have that people skill.” [Ontario, Participant #6].*

Participants also varied in their views on the involvement of family members in these conversations. Some individuals preferred having a family member present during this conversation (for support, to help make decisions, for transparency, and to remember what was said), while some cited family dynamics as potentially disruptive, and others explicitly stated that they wished to be alone with their physician. In cases where a patient may be experiencing cognitive impairment, the involvement of a family member or caregiver was regarded as necessary. Overall, participant responses indicated the need for an individualized approach.

*“So yes, I think that it needs, it needs to be done subtlety but I don’t believe in, oh well because if they have a gentile nature, we won't tell them that they are dying or that they only have 3 months to go. I want to know that I have 3 months to go.” [Nova Scotia, Participant #1]*

*“In my case, my family members know everything about me anyway. And then they’d understand what the doctors talking about. So things are made clear on all sides.”[Ontario, Participant #1]*

The main challenge with informing individuals that they are at risk of deteriorating health or death is that not all individuals want to be reminded of death, nor are they comfortable talking about it. Therefore, the potential exists for patients to become emotionally distressed. Where the patient has a good relationship with their physician, this may be mitigated.

“*I still think that there will be a few people who will just say ‘no’ and don’t want to hear anything about it. I may be wrong, but I know that there are people who don’t want to be told when they are terminally ill or anything like this…my late husband being one of them…he just wouldn’t talk about death because that kind of made it sound as though he was going to die*.” [*Nova Scotia, Participant #3*]

### Supporting patients and families

Where supports relevant to the conversation itself were identified, they included informational and emotional supports. Participants felt physicians should provide patients with sufficiently detailed information about their medical status and resources available within the community in order to start planning next steps.

*“I’m there to fish their brains for what they know that I don’t. And for them to pass it on to me so I can make decisions in my life. And I think their responsibility is to pass the information on to the patient as best they can and as honestly as they can so that the patient realizes that I have to make some decisions for my future and what I’m going to do.” [Nova Scotia, Participant #6]*

Other specific informational supports that were identified included educational materials about ACP, access to information on writing and updating wills, power of attorney, samples of advance care plans, and a contact number that could be called if questions arose at a later date.

*“Wills that had not been updated in a long time. Making sure that our powers of attorneys had been updated. And ah funeral – funeral arrangements or whatever you think about that. Because by having that all done in advance it made it so much easier on the family when the time came.” [Ontario, Participant #8].*

The term “emotional support” was not used by the participants themselves, however, when asked what would make them feel supported during conversations about ACP, participants identified a variety of strategies that their physician might employ that would address various emotional needs. For example, participants expressed wanting to feel that they were being listened to, that their values and preferences were being respected, that their input was valued, and that the physician was acting in the best interest of the patient.

## Discussion

This study explored patient and caregiver perspectives with regard to the identification of individuals at risk of deteriorating health or death earlier on in their disease trajectory in PHC settings, and the initiation of ACP by PCPs. Participants in our study regarded ACP favorably and agreed that the early identification of at-risk individuals for the purposes of ACP initiation was appropriate. They were also supportive of the use of a computerized algorithm to assist with early identification, although limitations were recognized. PHC was generally perceived as a suitable setting for both early identification and ACP initiation to occur.

In order for ACP to be meaningful, individuals at risk of dying must be identified earlier in their disease trajectory, so that discussions can take place to support the development and implementation of advance care plans that align with the needs and wishes of the patient. Unfortunately, physicians have consistently reported hesitancy to initiate conversations related to planning for EOL based on the perception it may be too upsetting or stressful for patients [44–46] or cause them to lose hope [33]. While some research has shown that some patients are in fact reluctant to discuss death and/or engage in ACP [33, 47], this is not always the case [48]. The majority of participants in our study viewed early identification in PHC settings positively, as a means of reducing stress for patients and families and allowing patients to focus on what matters most to them, although there was also recognition that discomfort with discussing death may be a barrier to ACP for some individuals. These findings, together with existing literature, suggest that physicians may be overestimating the negative impacts of raising the topic of ACP with patients.

One of the reported barriers to ACP is that providers have difficulty identifying patients who are at risk of dying in the near future [23, 24]. As such, the identification of those approaching the EOL subsequently occurs too late for proactive needs and desired care plans to be put into place and carried out [24]. To facilitate early identification, we explored the use of an algorithm based on EMR data to routinely and systematically identify patients who are at risk of deteriorating health or death in order to trigger the initiation of ACP. While the feasibility of this approach has been demonstrated elsewhere by Mason et al. [49], similar efforts have faced issues related to public perception. Specifically, efforts in the United Kingdom to assist PCPs in identifying patients expected to die within 1 year [36] were met with claims from the media that ‘death lists’ were being created and for the purposes of rationing health care, which caused unnecessary distress among patients and families [50, 51]. The participants in our study did not express similar concerns, which may be indicative of different societal attitudes about the use of EMR data, but may also be related to the fact that they received substantial information about the study and its aims prior to completing the interview, and therefore understood that the intended aim of the algorithm was to improve patient care. In other words, the interview itself may have served as an intervention and the views expressed may not accurately reflect how individuals would react to the use of an EMR-based algorithm and/or being identified as at risk of death. In practice, the implementation of an EMR-based algorithm to identify individuals as being at risk of death in PHC settings must be accompanied by a communication strategy aimed at the general public that clearly communicates the objectives of the routine and systematic identification of individuals at risk of death: to facilitate ACP initiation and ensure patient-centered care as individuals approach the end of life. Such a campaign would be integral to maintaining trust between patients and physicians, and mitigating negative media attention.

Regarding ACP conversations, our findings show that individual preferences vary greatly with respect to the style of approach, explicit mention of death or dying, involvement of family members, and the type of supports required. This is consistent with other studies [47, 52] that have reported highly personalized ACP preferences, and highlights the need for an approach that is tailored to each individual patient. This also points to the value of having ACP led by someone who is familiar with the patient and has established a rapport with them. Participants were generally in agreement that having their PCPs initiate ACP would be appropriate, but questioned the feasibility of having them take on this role given physicians’ workload and time constraints—challenges that have been echoed by physicians themselves [40, 53]. In a companion study by the authors [40], stakeholders (including 29 health care providers) felt that while ACP is incredibly important, physicians do not have the time to engage in the multiple, in-depth discussions required. This issue may be related to the fact that many family physicians work in solo practices or physician-only practices without other professionals (e.g., nurses, nurse practitioners, social workers, etc.) to help support this work. Perceived lack of time may also be related to how physicians are compensated for the care they provide. The majority of family physicians in Canada are paid through a fee-for-service model [54], which promotes shorter visits. Moreover, not all provincial/territorial fee schedules include codes for ACP, meaning physicians in some jurisdictions cannot be compensated for ACP. Time availability and funding/reimbursement have also been identified as barriers to advance care planning in primary care settings in other jurisdictions [55, 56]. An important step to increasing ACP, particularly in primary care settings, is ensuring that funding mechanisms are in place to encourage providers to the take the necessary time to meaningfully engage in ACP with patients and family/friend caregivers [7, 57].

There are several limitations to the work presented here. First, this study included patients and family/friend caregivers from only two provinces (Nova Scotia and Ontario) in Canada. Secondly, not all participants had personal experience with or a detailed understanding of ACP prior to their participation in the study. For some individuals, this study was the first time they had heard of ACP or been asked to reflect on their preferences; thus, their responses may have been influenced by the definition of ACP that the research team provided. Similarly, the EMR-based algorithm was not developed at the time of this study, so participants were asked to respond based on a description of the algorithm provided by the research team. Nonetheless, the findings reported here provide unique insights for improving the capacity of PCPs to initiate ACP with patients who may benefit from it.

## Conclusions

Overall, the findings of this study are supportive of practice-level identification of individuals at risk of deteriorating health or death and point to the general acceptability of using an EMR-based algorithm as a tool to facilitate the systematic and early identification of these individuals. The work related to algorithm development and validation remains ongoing. Participants’ responses support the continuation of this work, however, the concerns expressed by participants about the use of an EMR-based algorithm (e.g., privacy, physician time constraints) point to important implementation considerations. ACP preferences varied substantially amongst participants, highlighting the importance of a patient-centered approach in all aspects of patients care, including the process of ACP development and implementation. The need for a personalized approach reinforces the notion that family physicians are well positioned to take on a greater role in ACP (i.e., based on their familiarity and frequent contact with the patient). At the same time, the time required to prepare patients for ACP and to effectively engage them in this process suggest that ACP should not be considered the responsibility of the physician alone; rather, other health care providers and allied health professionals should also be engaged in efforts to increase ACP uptake in Canada. To improve ACP capacity in primary care settings, compensation systems for PCPs should be adjusted to ensure appropriate compensation and to accommodate longer ACP appointments, which would allow them to be more responsive to individual patient needs. Additional resources and more established links to community organizations and services must also be established to facilitate referrals to relevant community services as part of the ACP process.

## Data Availability

The datasets generated and/or analyzed during the current study are not publicly available due to the possibility that participant privacy may be compromised, but are available from the corresponding author on reasonable request.

## Interview Guide

### About advance care planning

1. If a patient wanted to have a conversation about advance care planning (ACP), who do you think is the best person to have these conversations with (e.g., primary health care provider, a specialist, nurse, social worker, etc.)? Why?
2. If the [individual(s) identified in #1] believes a patient is at risk of deteriorating health and that they may benefit from ACP,
  - How should they bring it up with the patient? (e.g., in-person conversations with your doctor at a scheduled appointment, let you know beforehand via telephone conversations or mail-out containing information, involve/not involve family or other supports, etc.)
  - Are there things they should avoid doing? If so, what are these?
  - What would you want them to say, or not say?
3. Can you describe to me a ‘best-case scenario’ in terms of how a family doctor, or other health team member, might initiate and support a person and his/her family through these discussions? Could you describe what you would want this process to be like for yourself or a loved one?
4. How could doctors and healthcare teams best support the person and their family members during these discussions?

### Views on Early Identification

5. As previously mentioned, ideally, ACP occurs while a patient is still well enough to make decisions and have discussions about their future health care.
  - What do you think about this idea of doctors identifying patients who are at risk of deteriorating health and who might benefit from advanced care planning?
  - Can you think of the benefits of doing this?
  - Can you think of any problems with doing this?
6. At one time, when you went to see a doctor, your information was collected in a paper chart. These days it is collected in an electronic medical record.
  - What are you views on your doctor using information contained within your electronic health record to determine whether you (or someone you love) is at risk of deteriorating health?
  - Do you see any benefits of your doctor using your electronic medical records?
  - Do you see any problems with this?
7. How do you think this idea of early identification should be messaged or communicated to make sure that patients, families, and the general public understand the benefits of this approach (e.g., in-person conversations with your doctor to prepare you in advance, telephone conversations, mail-out containing information, posters in your doctor’s office, ads on television/radio, etc.)?

### Interventions

8. What things would best support seriously ill patients and their families once they have been identified as being at risk of deteriorating health (e.g., information available to take home regarding ACP, contact information, community supports, follow up appointments, referrals to other professionals such as social workers, legal aids, etc.)?
9. What things would help make sure that a person’s goals for future care are met? For example, a patient’s goal might be to stay in their own home. What sort of things would be required to achieve that goal? What other goals might a person have and how could health professionals help them achieve those?

### Conclusion

10. Is there anything at all we have not talked about that you believe is important to understand about ACP, or earlier identification of persons at risk of deteriorating health? If so, what?

## Abbreviations

ACP: Advance care planning
CHF: Congestive heart failure
COPD: Chronic obstructive pulmonary disease
EMR: Electronic medical record
EOL: End-of-life
PCP: Primary care provider
PHC: Primary healthcare
